# Disrupted Topological Organization of Resting-State Functional Brain Networks in Age-Related Hearing Loss

**DOI:** 10.3389/fnagi.2022.907070

**Published:** 2022-05-20

**Authors:** Wei Yong, Jiajie Song, Chunhua Xing, Jin-Jing Xu, Yuan Xue, Xindao Yin, Yuanqing Wu, Yu-Chen Chen

**Affiliations:** ^1^Department of Radiology, Nanjing First Hospital, Nanjing Medical University, Nanjing, China; ^2^Department of Radiology, Nanjing Pukou Central Hospital, Pukou Branch Hospital of Jiangsu Province Hospital, Nanjing, China; ^3^Department of Otolaryngology, Nanjing First Hospital, Nanjing Medical University, Nanjing, China; ^4^Department of Otolaryngology, Nanjing Pukou Central Hospital, Pukou Branch Hospital of Jiangsu Province Hospital, Nanjing, China

**Keywords:** age-related hearing loss, brain function, functional magnetic resonance imaging, graph theory, small-world network

## Abstract

**Purpose:**

Age-related hearing loss (ARHL), associated with the function of speech perception decreases characterized by bilateral sensorineural hearing loss at high frequencies, has become an increasingly critical public health problem. This study aimed to investigate the topological features of the brain functional network and structural dysfunction of the central nervous system in ARHL using graph theory.

**Methods:**

Forty-six patients with ARHL and forty-five age, sex, and education-matched healthy controls were recruited to undergo a resting-state functional magnetic resonance imaging (fMRI) scan in this study. Graph theory was applied to analyze the topological properties of the functional connectomes by studying the local and global organization of neural networks.

**Results:**

Compared with healthy controls, the patient group showed increased local efficiency (E_loc_) and clustering coefficient (C_p_) of the small-world network. Besides, the degree centrality (Dc) and nodal efficiency (Ne) values of the left inferior occipital gyrus (IOG) in the patient group showed a decrease in contrast with the healthy control group. In addition, the intra-modular interaction of the occipital lobe module and the inter-modular interaction of the parietal occipital module decreased in the patient group, which was positively correlated with Dc and Ne. The intra-modular interaction of the occipital lobe module decreased in the patient group, which was negatively correlated with the E_loc_.

**Conclusion:**

Based on fMRI and graph theory, we indicate the aberrant small-world network topology in ARHL and dysfunctional interaction of the occipital lobe and parietal lobe, emphasizing the importance of dysfunctional left IOG. These results suggest that early diagnosis and treatment of patients with ARHL is necessary, which can avoid the transformation of brain topology and decreased brain function.

## Introduction

With the increasingly serious aging of the world population, age-related hearing loss (ARHL), the third most common disease of the elderly, has attracted more and more attention (Loughrey et al., 2018; Slade et al., 2020). ARHL, associated with the function of speech perception decreases, is characterized by bilateral sensorineural hearing loss at high frequencies function (Lee et al., 2010; Rutherford et al., 2018; Sharma et al., 2021). The function of speech perception decreases shows slowed central processing of acoustic information in noisy environments (Gates and Mills, 2005; Yamasoba et al., 2013; Li et al., 2017). These problems not only contribute to the seriously decreased quality of life but also lead to social isolation and falls and shorten the life span of patients (Kamil et al., 2016). However, less is known about the exact neuropathological mechanism of ARHL and its relationship with cognitive impairment.

Although impaired inner ear function is the main cause of ARHL, it is increasingly recognized that ARHL is also related to structural and functional changes in the central auditory pathway and other areas of the central nervous system (Kazee et al., 1995; Spongr et al., 1997; Salvi et al., 2002; Ouda et al., 2015). With the further application of functional magnetic resonance imaging (fMRI) which was based on blood oxygen level dependent (BOLD) in central nervous system abnormalities of ARHL, some studies have found that hearing loss devoted to the disrupted functional networks such as limbic network (SCLN), default mode network (DMN), executive control network (ECN), attention network (AN), and visual network (VN) (Chen et al., 2018, 2020; Xing et al., 2020, 2021a,b; Ren et al., 2021). In addition, experiments have found abnormal structural and functional visual centers similar to the auditory center in ARHL (Schulte et al., 2020; Wei et al., 2021), proving that the dysfunction caused by hearing loss involves the whole brain (Benetti et al., 2021).

Recent studies using magnetic resonance spectroscopy (MRS) have shown decreased neurotransmitters such as gamma-aminobutyric acid (GABA) in ARHL related to age and speech in noise, indicating that the reduction of neurotransmitters in the auditory system is related to functional impairment (Gao et al., 2015; Dobri and Ross, 2021). Many fMRI studies and animal experiments have linked the decline of cognition with functional abnormalities in ARHL, as well as dementia and depression (Chen et al., 2020; Choi et al., 2021; Ren et al., 2021; Shen et al., 2021). However, the causal relationship between the degeneration of peripheral auditory system, such as inner ear structure, and the declined function of central auditory system and cognitive function in patients has not been clear (Rutherford et al., 2018; Bowl and Dawson, 2019; Ralli et al., 2019).

Graph theory provides a theoretical framework for analyzing the topology of brain networks by studying the local and global organization of neural networks. At present, it has been widely used to study the properties of complex networks (Lv et al., 2018; Sporns, 2018; Hallquist and Hillary, 2019). In the graph theory model, the human brain is characterized as a large-scale network consisting of nodes and edges, defined brain regions as nodes while edges as an anatomical connection or functional correlation between two nodes (Medaglia, 2017). The brain network can be divided into different modules to separate functionally related neurons and observe the connection and flow of information. These modules not only complete different functions independently but also participate in the integration of whole brain function jointly through the core nodes. Interestingly, the information transmission of our brain network reflects low cost and efficiency. It exhibits characteristics of the small-world network, which means small networks of highly connected nodes in clusters with a few connections working together to carry out specific tasks or perform specific cognitive function (Van Den Heuvel et al., 2008; Wang et al., 2012).

Some specific properties of graph theory include characteristic path length, clustering coefficient, node degree and degree distribution, centrality, and modularity (Sporns et al., 2004; Reijneveld et al., 2007; Stam and Reijneveld, 2007), which can provide important new insights into the structure and function of networked brain systems including structure, development, and diseases. Therefore, in this study, we first used resting-state fMRI to construct the brain functional networks of patients with ARHL and analyze the topological features of their brain networks using graph theory.

## Materials and Methods

### Subjects

**Figure 1:**
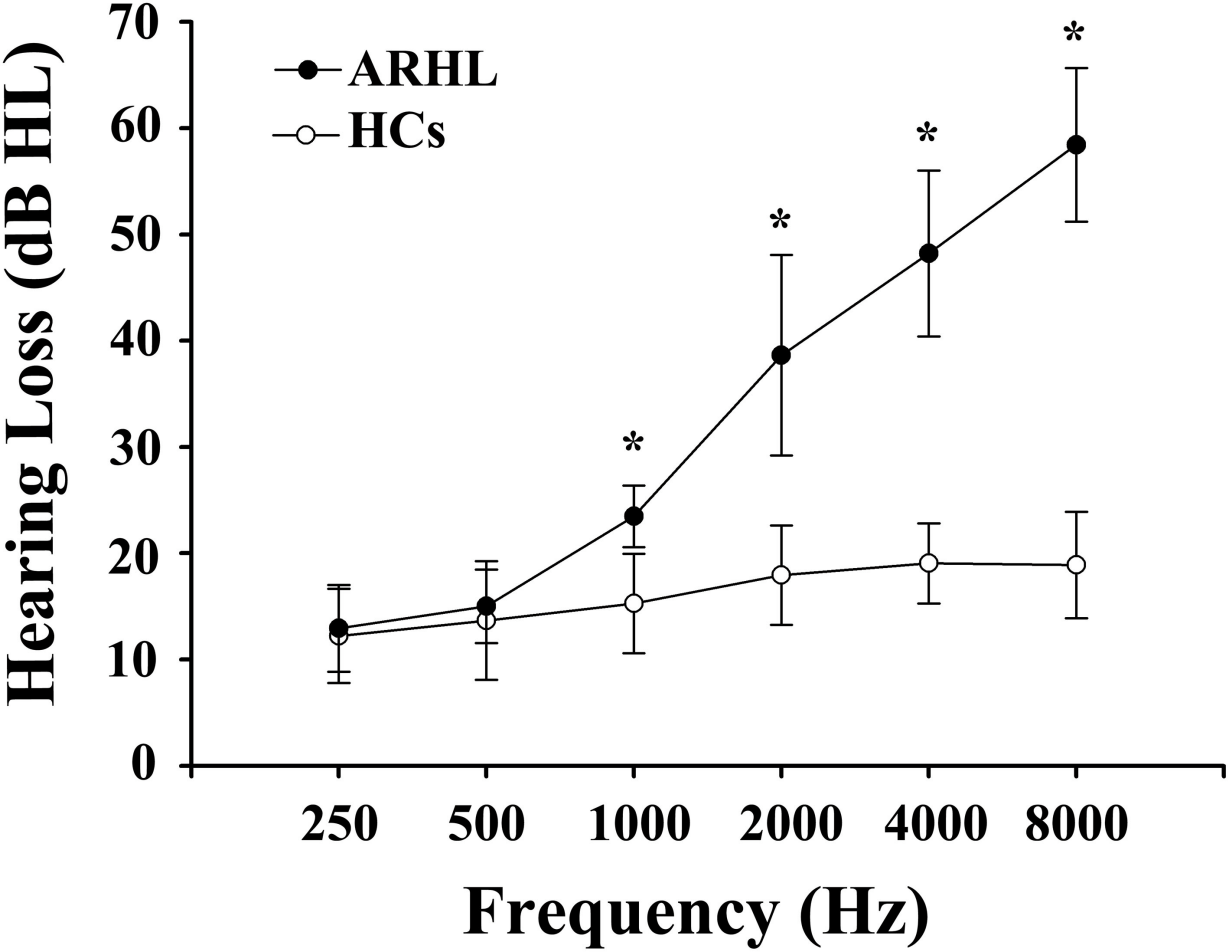
Mean hearing thresholds of age-related hearing loss (ARHL) patients and healthy controls (HCs). The hearing thresholds were significantly higher in ARHL than HCs (*p < 0.001, 1,000–8,000 Hz). Data are presented as mean ± SD.

We recruited 91 subjects (all right-handed and educated for at least 8 years) through community health screening and newspaper advertisements, including 46 ARHL patients and 45 age, sex, and education-matched healthy controls (HCs). Hearing loss was assessed by the speech-frequency pure tone average (PTA) of thresholds at the frequencies of 0.25, 0.5, 1, 2, 4, and 8 kHz. The PTA value of 25 dB HL was accepted as the normal hearing threshold limit. Inclusion criteria of the ARHL were average PTA > 25 dB HL in the better hearing ear and age ≥50 years. Tympanometry was performed with a Madsen Electronics Zodiac 901 Middle Ear Analyzer (GN Otometrics) to confirm normal middle-ear function. A summary of the mean hearing thresholds of both ears in all subjects is shown in Figure 1.

Exclusion criteria were ear diseases that affect hearing thresholds, including tinnitus, hyperacusis, and Meniere's diseases. To minimize the potential confounding factors, the following factors were excluded: ototoxic drug therapy, otologic surgery, noise exposure, alcoholism, brain injury, stroke, Alzheimer's disease, Parkinson's disease, major depression, epilepsy, neurological or psychiatric disorders that could affect cognitive function, major medical illness (e.g., anemia, thyroid dysfunction, and cancer), MRI contraindications, or severe visual loss.

All subjects underwent a battery of neuropsychological tests. The neuropsychological status of the subjects was established using the Mini Mental State Exam (MMSE), Montreal Cognitive Assessment (MoCA), auditory verbal learning test (AVLT), complex figure test (CFT), digit span test (DST), trail-making test (TMT) A and B, clock-drawing test (CDT), verbal fluency test (VFT), digit symbol substitution test (DSST), Self-Rating Anxiety Scale (SAS), and Self-Rating Depression Scale (SDS). It took about 1 h for each subject to complete all of the tests in a fixed order.

All the subjects provided written informed consent before their participation in the study protocol. Approval for the study was obtained from the Research Ethics Committee of Nanjing Medical University.

### MRI Acquisition

A 3.0 T MRI scanner (Ingenia, Philips Medical Systems, Netherlands) with an 8-channel receiver array head coil was used to scan. Foam padding and earplugs were used to reduce head motion and scanner noise. The subjects were required to close their eyes, lie down quietly, stay awake, not think about anything special, and avoid any head motion during the scan. Structural images were acquired with a three-dimensional turbo fast echo (3D-TFE) T1WI sequence with high resolution as follows: repetition time (TR)/echo time (TE) = 8.1/ 3.7 ms; slices = 170; thickness = 1 mm; gap = 0 mm; flip angle (FA) = 8°; acquisition matrix = 256 × 256; field of view (FOV) = 256 mm × 256 mm. The structural sequence took 5 min and 29 s. Functional images were obtained axially using a gradient echo-planar imaging sequence as follows: TR = 2,000 ms; TE = 30 ms; slices = 36; thickness = 4 mm; gap = 0 mm; FOV = 240 mm × 240 mm; acquisition matrix = 64 × 64; and FA = 90°. The fMRI sequence took 8 min and 8 s.

### Data Preprocessing

Data preprocessing used Statistical Parameter Mapping 12 (http://www.fil.ion.ucl.ac.uk/spm) and the Graph Theoretical Network Analysis Toolbox for Imaging Connectomics (2.0.0A http://www.nitrc.org/projects/gretna/) (GRETNA). The processing pipeline included the following stages: (1) Removing the first 10 volumes because of patients to adjust to the environment and signal adjustment from the MRI. (2) Slice timing, corrected and realigned, was performed for the remaining 220 images. Any subjects with a head motion >2.0 mm translation or a 2.0° rotation in any direction were excluded. (3) The remaining dataset was normalized to the 3D-T1 data by the diffeomorphic anatomical registration through exponentiated lie algebra methods (reslicing voxel size as 3 × 3 × 3 mm 3). (4) Detrending and filtering (0.01–0.08 Hz) were performed in turn. Subsequently, several nuisance signals were regressed from the data including head motion, the global mean, and signals from white matter and the cerebrospinal fluid.

### Functional Connectivity Matrix and Graph Construction

The GRETNA software was used to construct the network (He et al., 2008; Zhang et al., 2011). First, automated anatomical labeling (AAL) atlas was applied to obtain 90 cortical and subcortical regions of interest in the whole brain, and each was taken for a network node(Tzourio-Mazoyer et al., 2002). Next, the mean time series was obtained for each region, and the partial correlations of the mean time series between all pairs of the nodes (representing their conditional dependencies by excluding the effects of the other 88 regions) were regarded as the edges of the network (Jin et al., 2011; Zhang et al., 2011; Tao et al., 2013). This process generated a partial correlation matrix (90 × 90) for each subject, which was converted to a binary matrix according to a predefined threshold. If the absolute partial correlation between regions *i* and area *j* exceeded the threshold, then entry a *ij* = 1; otherwise, a *ij* = 0.

The networks of individual subjects were different in the number of edges (Wen et al., 2011). To resolve this discrepancy, a range of sparse thresholds S to the correlation matrix was used to ensure that each graph had the same number of edges. For each participant, S was defined as the fraction of the total number of edges remaining in the network. The minimum value of S was set so that the average node degree of the threshold network was 2log(*N*), where *N* was the number of nodes. The threshold range generated by this process was 0.06 S 0.4, and the interval was 0.01. The networks generated by this threshold strategy could estimate the sparse properties of small-worldness and the smallest possible number of false edges (Watts and Strogatz, 1998; Zhang et al., 2011). For the brain networks at each sparsity level, we calculated both the global and node network parameters.

### Brain Functional Network Analysis

For the brain function network, the global topological structure of the brain function network and the regional properties of each node were characterized by calculating the global network parameters and the regional node parameters. The node parameters examined included Bc (betweenness centrality), Dc (degree centrality), Ne (nodal efficiency), nodal clustering coefficient, nodal local efficiency, and nodal shortest path. The global parameters examined included small-world parameters including L_p_ (characteristic path length), C_p_ (clustering coefficient), γ (normalized clustering coefficient), λ (normalized characteristic path length), and δ (small-worldness), and the network efficiency parameters included E_glob_ (global efficiency) and E_loc_ (local efficiency) (Watts and Strogatz, 1998; Eisensehr et al., 2001).

### Statistical Analysis

We calculated the area under the curve (AUC) for each network metric. The AUC for a general metric ⋎ was calculated over the sparsity range from S_1_ to S_n_ with an interval of ΔS, here *S*_1_ = 0.10, *S*_n_ = 0.34, and Δ*S* = 0.01. The AUC provided a summarized scalar for the topological characterization of brain networks, which is independent of a single threshold selection and sensitive to topological alterations in brain disorders (Wang et al., 2009; Zhang et al., 2011). The AUC value of each global parameter in the two groups, as a comprehensive evaluation of the index, was compared by a two-sample *t*-test. *p* < 0.05 was statistically significant. The Bonferroni correction was used for multiple brain regions between the two groups in node parameters.

For modular analysis, the network and node module in metric comparison of Gretna software was used to compare the functional connections within each module and between any two modules by a two-sample *t* test. The whole brain network is divided into six sub-modules, namely, the frontal lobe module, prefrontal lobe module, subcortical module, temporal lobe module, occipital lobe module, and parietal lobe module. The Bonferroni correction was used for multiple comparison correction. *p* < 0.05 was statistically significant. In addition, SPSS 19.0 statistical software was used to analyze the Spearman's correlation between the functional connection in or between modules and the global and node parameters. *p* < 0.05 was statistically significant.

## Results

### Demographic and Cognitive Characteristics

The demographic characteristics of ARHL and HCs were presented in Table 1. We observed no significant differences between the ARHL group and HCs in terms of age, sex, and education level. Besides, no significant difference was revealed in PTA between the left and right ear of the ARHL patients and HCs. The cognitive status of both groups was summarized in Table 2. Patients with ARHL performed significantly poorer on TMT-B score (*p* < 0.05). Significant differences in other cognitive performances were not observed.

**Table 1:** Demographics of the ARHL and HCs.

	**ARHL (*n =* 46)**	**HCs (*n =* 45)**	***p-*value**
Age (year)	62.657 ± 0.45	61.273 ± 0.71	0.264
Sex (male: female) Education level (years) PTA (Left, dB HL)	21/25 10.742 ± 0.03 33.034 ± 0.18	21/24 10.671 ± 0.68 16.262 ± 0.92	0.853 0.991 <0.001^*^
PTA (Right, dB HL) PTA (Both, dB HL)	33.656 ± 0.38 33.043 ± 0.88	16.093 ± 0.27 16.182 ± 0.34	<0.001^*^ <0.001^*^

*Data are represented as mean±SD*,

* *p-value <0.001*.

*ARHL, age-related hearing loss; HCs, healthy controls; PTA, puretone audiometry*.

**Table 2:** Neuropsychological scores of the ARHL and HCs.

	**ARHL (*n =* 46)**	**HCs (*n =* 45)**	***p*-value**
MMSE	28.891 ± 0.30	28.841 ± 0.30	0.864
MoCA	25.701 ± 0.70	26.221 ± 0.80	0.154
AVLT	33.597 ± 0.53	35.477 ± 0.29	0.230
CFT	34.451 ± 0.71	34.641 ± 0.58	0.566
CFT-delay	16.843 ± 0.53	17.283 ± 0.64	0.559
TMT-A	69.702 ± 0.97	68.622 ± 1.29	0.809
TMT-B	175.005 ± 1.21	153.474 ± 9.39	0.044^*^
CDT	3.480 ± 0.55	3.530 ± 0.55	0.633
DST	11.151 ± 0.59	11.822 ± 0.17	0.096
VFT DSST	14.374 ± 0.05 69.917 ± 0.94	15.303 ± 0.64 69.049 ± 0.90	0.252 0.645
SAS	36.835 ± 0.93	35.936 ± 0.59	0.499
SDS	38.599 ± 0.06	37.028 ± 0.41	0.396

*Data are represented as mean ± SD*,

* *p <0.05*.

*ARHL, age-related hearing loss; HCs, healthy controls; MMSE, Mini Mental State Exam; MoCA, Montreal Cognitive Assessment; AVLT, auditory verbal learning test; CFT, complex figure test; DST, digit span test, TMT-A, trail making test-Part A; TMT-B, trail making test-Part B; CDT, clock drawing test; VFT, verbal fluency test; DSST, digit symbol substitution test; SDS, Self-Rating Depression Scale; SAS, Self-Rating Anxiety Scale*.

### Modular Analysis

**Figure 2:**
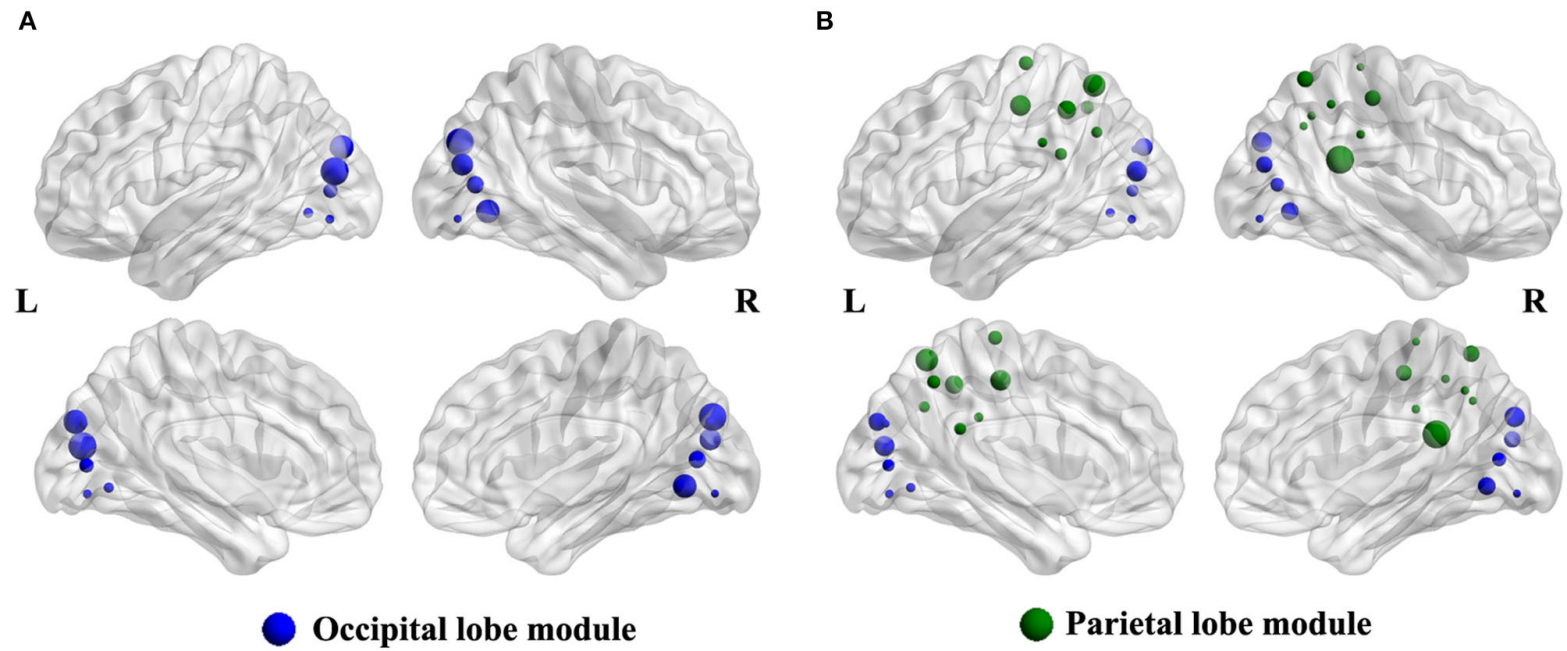
**(A)** The intra-modular interaction of occipital lobe module decreased in the patient group (*p* = 0.002). **(B)** The inter-modular interaction of parietal occipital lobe module decreased in the patient group (*p* < 0.001).

Compared with the HCs group, the intra-modular interaction of the occipital lobe module decreased in the patient group (*p* = 0.002, Bonferroni correction) (Figure 2A). Besides, the inter-modular interaction of the parietal occipital lobe module also decreased in the ARHL group (*p* < 0.001, Bonferroni correction) (Figure 2B).

### Nodal Level Analysis

**Figure 3:**
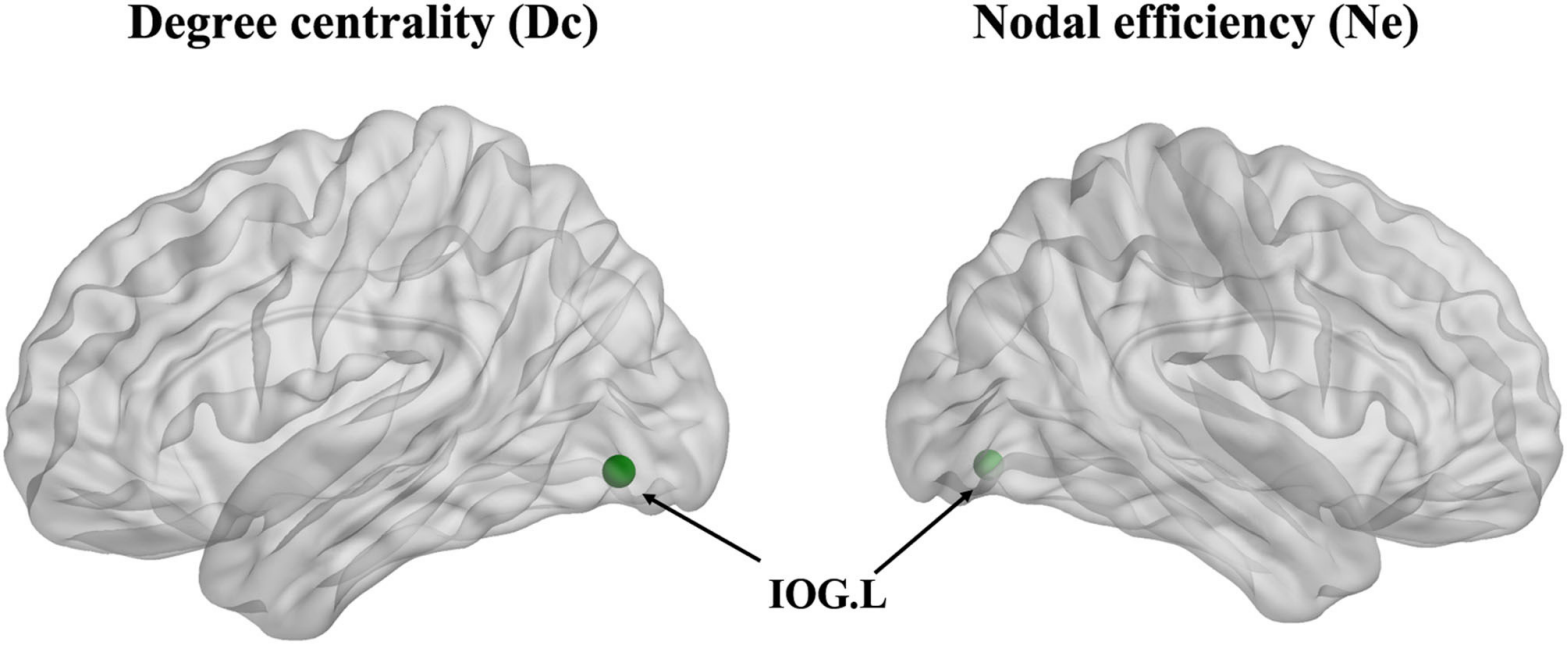
The degree centrality (Dc) and nodal efficiency (Ne) of the left inferior occipital gyrus (IOG.L) in the ARHL patient group showed a decrease compared with the healthy controls group (*p* < 0.001).

The degree centrality (Dc) of the left inferior occipital gyrus (IOG) in the patient group showed a decrease in contrast with the HCs (*p* < 0.001, Bonferroni correction) (Figure 3). Furthermore, the nodal efficiency (Ne) of the left IOG in the patient group showed a decrease in contrast with the HCs (*p* < 0.001, Bonferroni correction) (Figure 3). However, the betweenness centrality (Bc) showed no differences between the two groups.

### Global Level Analysis

**Figure 4:**
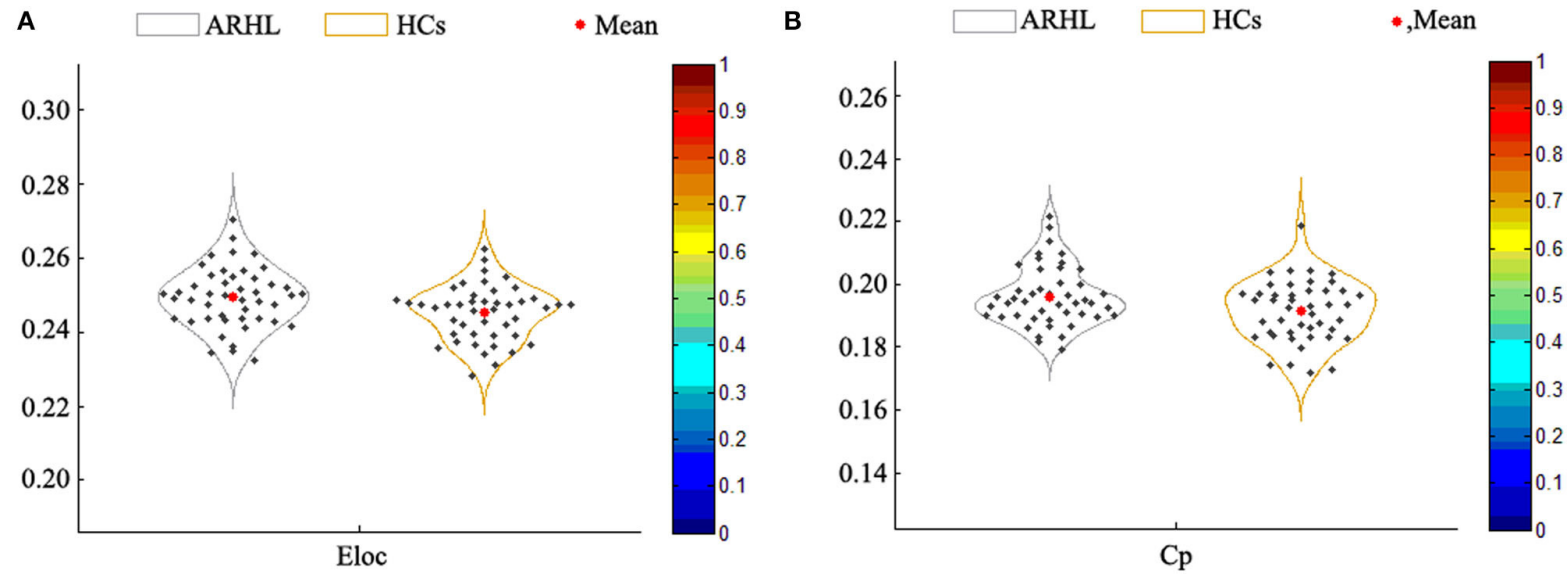
**(A)** The local efficiency (E_loc_) of the patient group was higher than the healthy controls (*p* = 0.013). **(B)** The clustering coefficient (C_p_) of the patient group was higher than the control group (*p* = 0.019).

Compared with the HCs group, the local efficiency (E_loc_) of the ARHL group was higher (*p* = 0.013, *p* < 0.05) (Figure 4A). But the global efficiency (E_glob_) showed no difference between the two groups. Otherwise, the clustering coefficient (C_p_) of the patient group was higher than the control group (*p* = 0.019, *p* < 0.05) (Figure 4B). As for other parameters including normalized clustering coefficient (γ), normalized characteristic path length (λ), the characteristic path length (L_p_), and small-worldness (σ), there were no difference between both groups.

### Correlation Analysis

**Figure 5:**
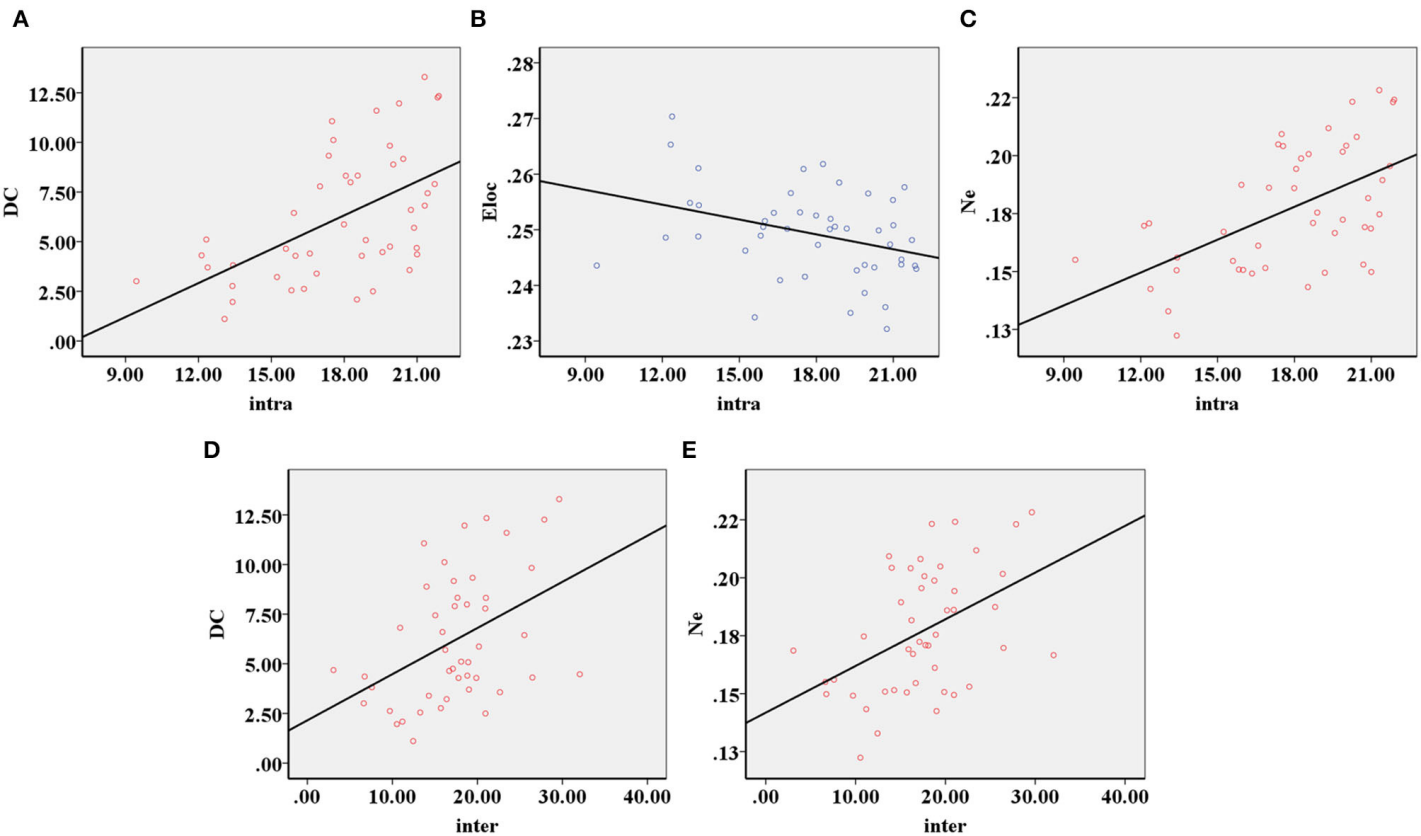
The correlation analysis of modular analysis with nodal and global parameters. **(A)** The decreased intra-modular interaction of the occipital lobe module in the patient group was positively correlated with the Dc (*p* < 0.001). **(B)** The decreased intra-modular interaction of occipital lobe module in the patient group was negatively correlated with the E_loc_ (*p* = 0.020). **(C)** The decreased intra-modular interaction of occipital lobe module in the patient group was positively correlated with the Ne (*p* < 0.001). **(D)** The decreased inter-modular interaction of parietal occipital lobe module in the patient group was positively correlated with the Dc (*p* = 0.003). **(E)** The decreased inter-modular interaction of parietal occipital lobe module in the patient group was positively correlated with the Ne (*p* = 0.001).

The decreased intra-modular interaction of the occipital lobe module and decreased inter-modular interaction of the parietal occipital lobe module in the ARHL group were positively correlated with the Dc (*p* < 0.001, *p* = 0.003) (Figures 5A,D) and Ne (*p* < 0.001, *p* = 0.001) (Figures 5C,E). The decreased intra-modular interaction of the occipital lobe module in the ARHL group was negatively correlated with the E_loc_ (*p* = 0.020) (Figure 5B). However, the decreased inter-modular interaction of the parietal occipital lobe module in the ARHL group showed no correlation with the E_loc_ (*p* = 0.056). Similarly, the decreased intra-modular interaction of the occipital lobe module and the decreased inter-modular interaction of the parietal occipital lobe module in the ARHL group also showed no correlation with the C_p_ (*p* = 0.301, 0.605).

## Discussion

The occipital lobe not only plays an important role in integrating information of visual sense, auditory sense, and other information gathered by sensory systems but also connects visual information with brain processing systems of speech and other executive functions (Wu et al., 2020). The parietal lobe is essential to process sensory information, including integration, perception, digital cognition, speech understanding, decision-making, and spatial consciousness (Critchleey, 1953; Bisley and Goldberg, 2010). We found that the intra-modular interaction of the occipital lobe module and the inter-modular interaction of the parietal occipital module decreased in the patient group, indicating the disrupted function of the occipital lobe and parietal lobe. This was similar with previous studies (Zhang et al., 2021). One of the reasons may be the cross-modal functional reorganization due to a variety of sensory processing abnormalities caused as a result of hearing loss (Luan et al., 2019; Wei et al., 2021). The cross-modal plasticity is an internal ability of the brain, which represents a compensation mechanism when a specific sensory pattern is deprived (Benetti et al., 2021). Conjectured in this experiment, parietal and occipital resources of ARHL are occupied to compensate for hearing loss. However, excessive occupied resources for a long time may cause the functional change or even decline of the parietal occipital lobe. Therefore, early diagnosis and treatment of ARHL is necessary to decrease the negative impact on other brain regions (Glick and Sharma, 2017).

The IOG participates in the related processes of visual processing such as correlated gradients of spatial and face-part selectivity due to typical face-directed gaze behavior (De Haas et al., 2021). As a simple measurement of connectivity between a single node and all other nodes in networks, the DC represents the importance of a node (Telesford et al., 2011). The NE measures how efficiently information is exchanged over the network (Ottet et al., 2013). The decrease of Dc and Ne of the left IOG was positively correlated with the intra-modular interaction of the occipital lobe module and the inter-modular interaction of the parietal occipital module, indicating that the left IOG is a core node for information transmission within the occipital lobe and between the parietal occipital lobe (De Haas et al., 2021). In other words, the dysfunction of the left IOG plays an important role in the integration of whole brain function and further affects the connection between the occipital lobe and the parietal lobe. It is suggested that the study of the dysfunction of occipital and parietal lobes in ARHL in the future should focus on the IOG. For the treatment of ARHL, the abnormality of occipital and parietal lobes may improve with the IOG targeted.

While a small-world network between them has smaller L_p_ and larger C_p_, which support the differentiation and integration of information with high efficiency (Van Den Heuvel and Hulshoff Pol, 2010). To a certain extent, the small-world network has the ability to resist disease attacks (Achard and Bullmore, 2007). The increased *C*_p_ represents the imbalance of differentiation and integration of the small-world network in the patient, which tends to the topology of a regular network and easier disease attacks. The information transmission speed of the regular network is lower than the random network in the brain level, which indicates that the topology transformation of the small-world network in ARHL may decrease the connectivity of the whole brain, and then lead to brain cognitive impairment (Van Den Heuvel and Hulshoff Pol, 2010; Lv et al., 2018). Therefore, early treatment of patients with hearing loss is necessary to prevent the transformation of topology and the decline of cognitive function.

The increase of C_p_ and E_loc_ represents the improvement of local network information processing efficiency of patients (Lv et al., 2018). We speculate that the decrease of DC and NE in the left IOG results in the decreased interaction within the occipital module and between the parietal occipital module, and the brain mobilizes more resources to solve this problem leading to improved local network information processing ability. Although this compensation mechanism can alleviate the dysfunction of local brain areas, the consumption of more resources may decrease the information processing ability of the global brain level, resulting in the abnormalities of other brain areas (Fornito et al., 2015). The transformation of small-world network topology may be the result of ARHL and the compensation mechanism. Interestingly, there was no difference in E_glob_ and L_p_ between the two groups, indicating no significant change in the information processing ability of the global brain level, which may be the cause of the pathogenesis of ARHL and insufficient sample size. On the contrary, it may be that sufficient brain information processing ability allows compensatory changes.

## Limitation

First, this experiment is a cross-sectional study. A small sample size may lead to inaccurate results. Second, although earplugs have been used, the noise during MRI scan would have a certain impact on this experiment. Finally, our interpretation of the results is subjective to a certain extent because of few articles on graph theory and ARHL. Sufficient samples and multiple experiments are essential for ARHL.

## Conclusion

Based on resting-state fMRI and graph theory, this experiment found decreased intra-modular interaction of the occipital lobe module and decreased inter-modular interaction of the parietal occipital lobe module. We prove the transformational topology of the small-world network in ARHL, which may cause the decline of global brain connectivity and brain cognitive impairment. These results suggest that early diagnosis and treatment of patients with ARHL is necessary, which can avoid the transformation of brain topology and decreased brain function. Our research suggested that the disorder of brain network topology may play a pivotal role in cognitive impairment of ARHL, which may be a potential imaging biomarker for early clinical diagnosis, prevention, and treatment of ARHL.

## Data Availability Statement

The original contributions presented in the study are included in the article/supplementary material, further inquiries can be directed to the corresponding author/s.

## Ethics Statement

The studies involving human participants were reviewed and approved by the Research Ethics Committee of Nanjing Medical University. The patients/participants provided their written informed consent to participate in this study.

## Author Contributions

WY and JS drafted the manuscript for the work. CX, J-JX, and YX helped to acquire the clinical and fMRI data. XY helped to revise the manuscript critically for important intellectual content. YW and Y-CC did the financial support, review, and final approval of the manuscript to be published. All authors have read and approved the final manuscript.

## Funding

This work was supported by the Natural Science Foundation of Jiangsu Province (No. BK20211008) and the Medical Science and Technology Development Foundation of Nanjing Department of Health (No. YKK21133).

## Conflict of Interest

The authors declare that the research was conducted in the absence of any commercial or financial relationships that could be construed as a potential conflict of interest. The reviewer JC declared a shared parent affiliation with the authors WY, JS, CX, J-JX, XY, YW, and Y-CC to the handling editor at the time of review.

## Publisher's Note

All claims expressed in this article are solely those of the authors and do not necessarily represent those of their affiliated organizations, or those of the publisher, the editors and the reviewers. Any product that may be evaluated in this article, or claim that may be made by its manufacturer, is not guaranteed or endorsed by the publisher.
